# Single cell multiomic analysis of the impact of Delta-9-tetrahydrocannabinol on HIV infected CD4 T cells

**DOI:** 10.1186/s42238-026-00412-0

**Published:** 2026-03-05

**Authors:** Manickam Ashokkumar, Renee Y. Ge, Alicia Cooper-Volkheimer, David M. Margolis, Quefeng Li, Yuchao Jiang, David M. Murdoch, Edward P. Browne

**Affiliations:** 1 Department of Medicine, University of North Carolina at Chapel Hill, Chapel Hill, North Carolina USA; 2 UNC HIV Cure Center, University of North Carolina at Chapel Hill, Chapel Hill, North Carolina USA; 3 Department of Biostatistics, University of North Carolina at Chapel Hill, Chapel Hill, North Carolina USA; 4 Department of Medicine, Duke University, Durham, NC USA; 5 Department of Statistics, Texas A&M University, College Station, Texas USA; 6 Department of Biology, Texas A&M University, College Station, Texas USA; 7 Department of Microbiology and Immunology, University of North Carolina at Chapel Hill, Chapel Hill, North Carolina USA

**Keywords:** HIV latency, Cannabis, Δ-9-tetrahydrocannabinol, Transcriptomics, Chromatin accessibility

## Abstract

Cannabis use is prevalent among individuals living with HIV in the United States, but the impact of cannabis exposure on the reservoir of latently infected cells that persists during antiretroviral therapy (ART) remains unclear. To address this gap, we analyzed the effect of Δ-9-tetrahydrocannabinol (THC) on primary CD4 T cells that were latently infected with HIV. We found that THC did not have a strong effect on baseline or latency reversing agent (LRA) stimulated HIV expression, or on expression of an activation marker (CD38). However, using an integrated multiomic single-cell analysis of genome-wide chromatin accessibility and gene expression, we observed altered expression of several hundred genes in HIV infected CD4 T cells after THC exposure, including transcriptional downregulation of genes involved in protein translation and antiviral pathways, indicating that THC suppresses innate immune activation in infected cells. Additionally, chromatin accessibility analysis demonstrated upregulated chromatin binding activity for the transcriptional regulator CTCF, and reduced activity for members of the ETS transcription factor family in infected cells after THC exposure. These findings provide insights into the mechanisms by which cannabis use could influence the persistence of HIV within cellular reservoirs and the molecular phenotype of latently infected cells. Further elucidation of the underlying mechanisms involved in THC-mediated changes to HIV infected cells, will lead to an improved understanding of the impact of cannabis use on the HIV reservoir.

**Supplementary Information:**

The online version contains supplementary material available at 10.1186/s42238-026-00412-0.

## Importance

Cannabis use is common among individuals living with HIV, but the long-term effects of cannabis use on the HIV reservoir are not yet studied completely. We employed advanced single cell technologies to reveal how cannabis components, specifically THC, influence HIV-infected immune cells and their pattern of gene expression. We found that, while THC doesn't reactivate virus in latently infected cells, it alters the molecular characteristics of these infected immune cells. These findings are important because they underscore how cannabis could regulate persistent infection in people living with HIV. Understanding these cellular changes in response to THC could be helpful for successful treatment for people living with HIV.

## Introduction

Despite successful treatment with antiretroviral therapy (ART), people with HIV (PWH) retain a persistent viral reservoir, which is the primary barrier to achieving an HIV cure. This reservoir is comprised of a pool of latently infected cells that is distributed throughout various CD4 T cell subsets and tissues. In latently infected cells, viral gene expression is downregulated, but infected cells can still produce viral RNA and proteins which can potentially trigger an immune response and inflammation in PWH on ART (Cohn et al. 2020). This persistently elevated immune activation can lead to a higher risk of non-AIDS comorbidities, including cardiovascular disease, cancer, kidney, liver, and neurologic diseases (Manuzak et al. 2018). Despite its importance, little is known about the latently infected reservoir due to the rare nature of the infected cells and the lack of a specific marker that allows purification and characterization of these cells.

Some evidence suggests that the immune system of PWH, as well as the size and nature of the reservoir, can be impacted by drugs of abuse. Cannabis (CB) use, in particular, is prevalent amongst PWH. We have recently reported that PWH on ART who use CB exhibit a trend towards a smaller intact HIV reservoir, as well as elevated frequencies of naïve T cells, and lower frequencies of activated and exhausted CD8 T cells (Falcinelli et al. 2023). Consistent with this observation, another recent report indicated that CB use can lead to a reduced HIV reservoir burden in tissues in PWH (Liu et al. 2024). Other evidence from humans includes a recent study reporting that cannabis use was associated with lower frequencies of activated (HLA-DR^+^CD38^+^) CD4^+^ and CD8^+^ T cells, and significantly lower frequencies of TNF-α^+^ B cells in PWH on ART (Manuzak et al. 2018; Min et al. 2023). Animal studies have also pointed to an effect of CB on HIV infection and infection associated inflammation, including a recent report demonstrating that CB exposure leads to reduced inflammation in the gut of SIV infected animals (Kumar et al. 2019). In a study conducted in a humanized mice model, researchers found that Δ−9-tetrahydrocannabinol (THC) exposure increased the HIV viral load and decreased the number of IFN-γ secreting cells (Roth et al. 2005). By contrast, another study reported that chronic administration of THC to SIV-infected rhesus macaques decreased viral load in cerebrospinal fluid (CSF) and plasma as well as mortality (Molina et al. 2011).

Cannabinoids are lipophilic molecules that have been shown to alter the functional activities of immune cells in vitro and in vivo. THC is the most abundant and primary psychoactive cannabinoid component of cannabis. THC binds to and activates two endogenous cannabinoid receptors, CB1 and CB2. CB1 is largely present in brain and neurons, and CB2, by contrast, is expressed in immune cells, including CD4 T cells, the primary cell infected by HIV (Nagarkatti et al. 2009; Cabral and Griffin-Thomas 2009; McCoy 2016). Cannabinoid receptors regulate a complex signaling cascade that can affect CB-exposed cells on several levels. At the molecular level, activation of cannabinoid receptors leads to closure of Ca^2+^ channels and opening of K^+^ channels, inhibition of adenylyl cyclase activity and activation of a signaling cascade that leads to activation of the MAPK pathway (Howlett et al. 2010; Zou and Kumar 2018) including extracellular signal-regulated kinase 1/2 (ERK1/2), c-Jun N-terminal kinase (JNK), and p38, that are involved in the regulation of cell proliferation, cell cycle control and cell death. Cannabinoid receptors can also regulate expression of CREB and the pro-inflammatory transcription factor NF-κB (Samson et al. 2003).

CBs have complex effects on immune cell populations, and transcriptomic analyses of the effect of CBs on human immune cells have shown numerous alterations to transcriptional pathways in uninfected CD4 T cells (Falcinelli et al. 2023). THC has been shown to suppress the activities of B and T lymphocytes, reduce the cytolytic activity of NK cells, inhibit the function and maturation of cytotoxic T lymphocytes (CTLs), and affect immune cell recruitment and chemotaxis to sites of infection (Cabral and Griffin-Thomas 2009). CBs are canonically thought to be immunosuppressive; they reduce T cell proliferation and IL-2 expression after TCR-mediated activation, as well as TNF-α production and leukocyte migration across the blood-brain barrier (Cabral and Griffin-Thomas 2009; Klein et al. 2003). Thus, the collective data suggest that THC inhibits the functional activities of a variety of immune cells, an outcome that is consistent with these compounds altering host resistance to infectious agents (Cabral and Griffin-Thomas 2009).

Though the immunomodulatory properties of THC have been studied previously, its effect on cells that are latently infected with HIV has not yet been investigated. Since ~ 50% of PWH use cannabis, it will be important to fully evaluate the impact of THC exposure on the HIV reservoir and its responsiveness to clinical latency reversal approaches. In this report, we use a primary CD4 T cell model of HIV latency and a single cell multiomic profiling approach to examine the impact of THC exposure on a population of latently infected cells. We find that, although THC does not have a strong impact on HIV gene expression and responsiveness to latency reversing agents (LRAs) in this system, THC modulates the expression and activity of a set of genes and transcription factors in infected cells that may impact their behavior in PWH on ART.

## Methods

### Cell culture

Total CD4 T cells were isolated from peripheral blood mononuclear cells (PBMCs) from HIV seronegative donors using the EasySep Human CD4 T Cell Isolation Kit (STEMCELL Technologies, catalog # 17,952) using negative selection, following the manufacturer’s protocol. Purity of CD4 T cells was determined by flow cytometry, which indicated high purity (> 97%). Cells (CD4 or PBMCs) were seeded at 1 million cells/mL in RPMI media (Gibco), 10% fetal bovine serum (FBS), penicillin/streptomycin and incubated overnight at 37˚C. A human embryonic kidney cell line that was used for transfection (HEK293T—ATCC; CRL11268) was maintained in DMEM complete medium (Gibco), supplemented with 10% FBS and penicillin/streptomycin.

### Primary CD4 T cell HIV latency model

Latently infected primary CD4 T cells were generated as described in our previous studies (Ashokkumar et al. 2025, Ashokkumar et al. 2024). Briefly, CD4 T cells from seronegative donors were activated with anti-CD3/CD28 beads (Life Technologies) at 1 M cells per mL for 3 days, then infected by spinoculation for 2 h with supernatant from HEK293T cells that had been transfected with a replication defective HIV reporter plasmid (NL4-3-△6-drEGFP-IRES-thy1.2), as well as plasmids encoding GagPol and Vesicular Stomatitis Virus G protein (VSV-G) (Kumar et al. 2019; Guan et al. 2023). Successful infection was confirmed by measuring GFP expression (typically 10–20% of cells GFP +) using flow cytometry followed by enrichment of GFP + infected cells by flow sorting using a FACSAriaIII (Becton Dickson). Enriched infected cells were then kept in culture for 3 weeks while they returned to a resting state and viral gene expression was downregulated. To recapitulate a more physiological cell composition in the culture and to preserve intercellular communication networks, the infected CD4 T cells and matched PBMCs were mixed at a 1:3 ratio to generate a mixed population of latently infected cells and PBMCs.

### Δ−9-tetrahydrocannabinol stimulation and LRA treatment

Latently infected primary CD4 T cells were stimulated with Δ−9-tetrahydrocannabinol (THC) at concentrations ranging from 0 μM to 10 μM for 6 h or empty vehicle (0.015% methanol). At 6 h post THC exposure, the cells were harvested, washed and quantified for viral GFP expression, CD38 cell activation marker, and CD4 surface marker expression by flow cytometry. In addition to the dose response stimulation with THC, we also quantified reactivation and cell activation by measuring GFP and CD38 in different CD4 T cell subsets with THC exposure followed by LRA stimulation by one of three different LRAs with different mechanisms of action—AZD5582 (non-canonical NF-κB agonist), prostratin (PKC agonist), and vorinostat (HDAC inhibitor) or control vehicle (DMSO).

### THC stimulation for single cell ATACseq and gene expression

HIV infected primary CD4 T cells pooled with PBMCs of the same donor were stimulated with THC at a concentration of 500 nM or control vehicle (methanol 0.015%). Cells were then combined with autologous PBMCs that had been thawed and rested for 24 h at 37 ^o^C at a ratio of 1:3 (CD4 T cells: PBMCs). Each experiment was carried out with three biological replicates (separate donors). At 0 h, 3 h, 6 h or 12 h post THC exposure, the cells were harvested for integrated single cell multiomic ATAC and gene expression library preparation.

### Nuclei isolation

Nuclei were isolated following the 10xGenomics protocol (CG000365 • Rev C) as described in our previous study (Ashokkumar et al. 2024). Cells were harvested, washed once with ice-cold phosphate buffered saline (PBS)−0.5% BSA containing 0.2 U/μL RiboLock RNase inhibitor (Thermo Fisher, cat. no. EO0382), counted and viability determined by Trypan blue exclusion (Thermo Fisher, cat. no.15250061). Dead cells were removed using a dead cell removal kit (Miltenyi Biotec, cat. no. 130–090–101). For each sample, 1 × 10^6^ cells were collected by centrifugation at 500 g, 4 °C, 5 min. Cells were then resuspended in 100 μL of ice-cold lysis buffer (10 mM Tris–HCl, pH 7.4, 10 mM sodium chloride, 3 mM magnesium chloride, 0.01% Tween-20 (Sigma, cat. no. 655205-250ML), 0.01% nonidet P40 substitute (Sigma, cat. no. I8896), 0.01% Digitonin (Promega, cat. no. G9441), 1 U/μl RiboLock RNase inhibitor, 1% BSA and 1 mM DTT) and incubated on ice for 3 min. The released nuclei were then washed with 1 mL ice-cold wash buffer (10 mM Tris–HCl, pH 7.4, 10 mM sodium chloride, 3 mM magnesium chloride, 0.01% Tween-20, 1% BSA, 1 mM DTT and 1 U/μL RiboLock RNase inhibitor) and nuclei were re-suspended in diluted nuclei buffer (1X nuclei buffer, 1 mM DTT, 1 U/μL RiboLock RNase inhibitor).

### Construction of integrated single cell ATACseq and gene expression libraries and sequencing

Nuclei samples were used to generate single cell ATAC-seq and RNA-seq libraries using a Chromium Single Cell Controller (10xGenomics, Pleasanton, CA) and a Single Cell multiome ATAC and gene expression kit (CG000338 • Rev E). Briefly, diluted nuclei suspensions with a target recovery of 10,000 nuclei were subjected to Tn5 transposition in bulk, followed by barcoding using GEM (Gel Beads-in-emulsion) beads. Silane magnetic beads were used to purify the barcoded products from the post GEM-RT reaction mixture. Barcoded ATAC fragments from the transposed DNA and barcoded, full-length cDNA from poly-adenylated mRNA were then pre-amplified by polymerase chain reaction (PCR) to facilitate library construction. The pre-amplified product was then used as input for both ATAC and gene expression (GEX) library construction. P5 and P7 indices were added to the pre-amplified transposed DNA for ATAC-seq library. cDNA amplification, enzymatic fragmentation followed by end repair, A-tailing, adaptor ligation, and PCR were performed to incorporate P5, P7, i7 and i5 sample indices, and TruSeq Read 2 (read 2 primer sequence) for gene expression libraries. The libraries were quantified using an Agilent Tapestation 4200 and the Qubit dsDNA High Sensitivity Assay Kit (Invitrogen, #Q33230). Pooled samples for the ATAC and RNA libraries were sequenced using paired-end, single-index (ATAC-seq) and dual index (RNA-seq) sequencing on a NextSeq 2000 instrument (Illumina). For GEX libraries, the read format was: Read 1–28 cycles; Read 2–90 cycles; i7–10 cycles; i5–10 cycles. For ATAC libraries the read format was: Read 1–50 cycles; Read 2–49 cycles; i7–8 cycles, i5–16 cycles. Paired-end reads of pooled libraries were demultiplexed prior to downstream analysis.

### scRNA-seq and scATAC-seq data processing

Data from the three separate donors were aligned to the reference genome (Human GRCh38 and HIV NL4-3-△6-drEGFP-IRES-thy1.2) using cellranger-arc v2.0.0. Aligned datasets were processed separately for QC metrics. Cells with high percentages of mitochondrial reads (> 20%), extreme RNA read counts (> 15,000–20000), and/or extreme ATAC fragment counts (> 50,000–70000) were removed. Genes and peaks not expressed/accessible in at least 0.5% of the cells were removed with the exception of the HIV genes. Doublet cells, identified by scDblFinder (Germain et al. 2021), were excluded from subsequent analysis. For read count normalization, we used sctransform by Seurat (Stuart et al. 2019) for scRNA-seq and term frequency-inverse document frequency (TF-IDF) by Signac (Stuart et al. 2021) for scATAC-seq. This was followed by principal component analysis (PCA, 50) and latent semantic indexing (LSI, 2–50) for dimension reduction, respectively. Cell-type labels were transferred from an existing curated and annotated PBMC reference dataset (Jiang et al. 2022); cells with maximum prediction scores less than 0.8 for cell-type label transferring were removed. For modality-specific processing, we built the unweighted K-nearest neighbor (KNN) and the weighted shared nearest neighbor (SNN) graphs, followed by UMAP visualization and clustering identification (Becht et al. 2019).

To account for batch effects between the three biological replicates in the scRNA-seq data, we used anchor-based CCA Integration (Guan et al. 2023) to produce a joint dimension reduction, which was further used for UMAP visualization and clustering. Based on the integrated RNA UMAP, we observed CD4 T cells in two main subclusters: one exhibiting HIV Expression (HIV + CD4 T) and one without HIV Expression (HIV- CD4 T). For analyses regarding the impact of THC on HIV infected cells, only cells from the HIV + CD4 HIV cluster were considered. Clusters containing less than 50 cells were removed. Within the scATAC-seq data, we integrate the LSI embeddings across the donors, followed by UMAP visualization and clustering. We obtained the position frequency matrices and annotated 633 TF-binding motifs from the JASPAR 2020 database (Fornes et al. 2020); we further applied chromVAR (Schep et al. 2017) to derive, for each TF, its motif deviation score, which measures the deviation in chromatin accessibility across the set of peaks containing the corresponding motif, compared with a set of background peaks.

For each cell-type cluster, we performed a nonparametric Wilcoxon rank sum test to identify differentially expressed genes (DEGs) and differentially accessible motifs between the THC- and THC + conditions. We used the normalized gene expression levels by sctransform and the motif deviation scores by chromVAR as input, and adopted false discovery rate (FDR) for multiple testing correction. For significant DEGs, we carried out a gene ontology (GO) enrichment analysis using Enrichr (Kuleshov et al. 2016).

## Results

### Impact of THC on CD4 T cell surface marker phenotype and HIV expression

**Fig. 1:**
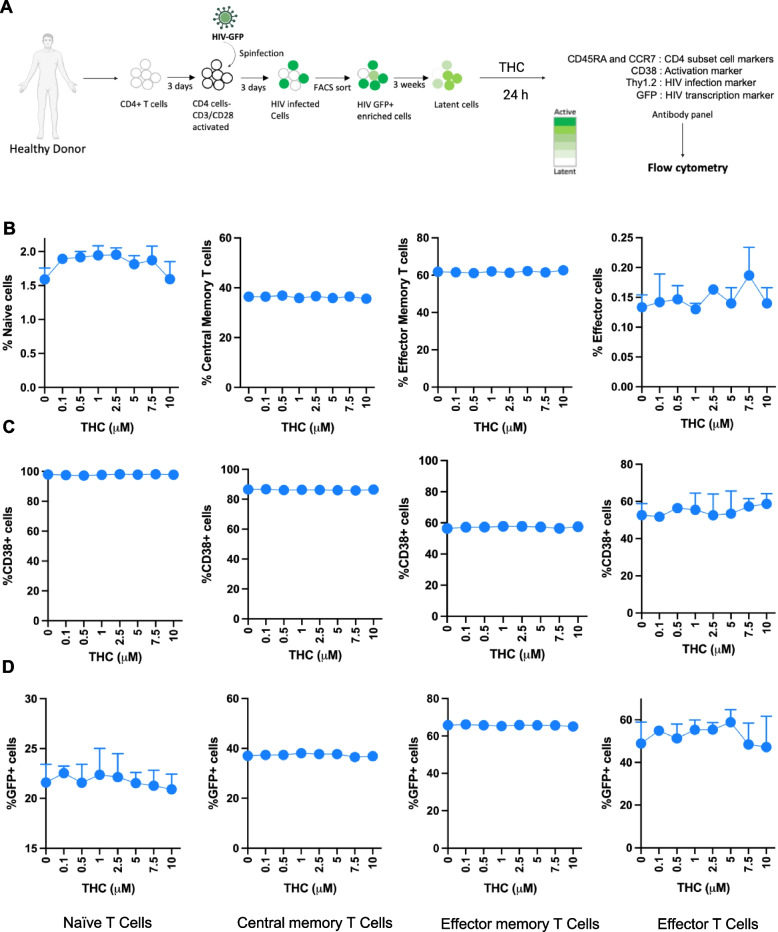
Impact of THC on cell surface marker phenotype and HIV expression in latently infected primary CD4 T cells. Latently infected CD4 T cells were exposed to THC at different concentrations for 24 h. At 24 h post exposure, we quantified the distribution of different CD4 T cell subsets, expression of activation marker, CD38, and HIV (GFP) expression. Cell subsets were assigned by expression of the phenotype surface markers, CD45RA and CCR7– Tn: CD45RA +/CCR7 +, Tcm: CD45RA-/CCR7 +, Tem: CD45RA-/CCR7-, Teff: CD45RA +/CCR7-. **A** Schematic of latently infected CD4 + T cell generation, pooling and THC stimulation followed by flowcytometry. **B** Abundance of CD4 T cell subsets—naïve (Tn), central memory (Tcm), effector memory (Tem) and effector (Teff) subsets at 24 h post THC administration/exposure. **C** Frequency of expression for the immune surface marker CD38 within CD4 T cells subsets (Tn, Tcm, Tem, and Teff cells). **D** Expression of eGFP (HIV) within CD4 T cells subsets (Tn, Tcm, Tem, and Teff cells). None of the samples achieved statistical significance relative to unexposed control cells

Although we and others have previously shown that cannabis (CB) use is associated with an altered abundance of T cell subsets and expression of activation markers in PWH on ART (Manuzak et al. 2018; Falcinelli et al. 2023), it is unknown if this reflects a direct impact of CB on the immune cells or on expression of the HIV reservoir. Therefore, we examined whether THC, the primary cannabinoid present in CB, affects the phenotype of HIV infected cells or expression of HIV using a primary CD4 T cell latency model that we have previously established. We added THC over a range of concentrations (0–10 μM) to ex vivo generated latently infected primary CD4 T cells (Fig. 1A). At 6 h and 24 h post stimulation with THC, flow cytometry was performed to determine the abundance of CD4 T cell subsets based on key surface markers (CCR7, CD45RA, and CD38), and HIV expression (GFP) (Figure S1). We first used CCR7 and CD45RA staining to assign cells as naïve (Tn—CD45RA +/CCR7 +), central memory (Tcm—CD45RA-/CCR7 +), effector memory (Tem—CD45RA-/CCR7-) or terminally differentiated effector cells (Teff—CD45RA +/CCR7-). We observed that, overall, in vitro THC exposure of latently infected primary CD4 T cells did not alter the frequency of cell subsets significantly at either timepoint (Fig. 1B, and Figure S2A). We then examined the impact of THC on expression of the surface marker CD38 in HIV infected CD4 T cells. CD38 is frequently used as a marker for activated CD4 T cells, but is also constitutively expressed in naïve and central memory CD4 T cells, where it regulates several aspects of CD4 T cell function, including regulating cellular NAD + levels (Camacho-Pereira et al. 2016). We observed no change in surface expression of CD38 for any of the subsets at any THC concentration (Fig. 1C, and Figure S2B). These data indicate that THC exposure does not have a detectable direct impact on the expression of CD4 T cell subset surface markers or on expression of CD38.

We then examined the impact of THC on viral gene expression (GFP) in infected cells (Fig. 1D and Figure S2C). In our latency model system, CD4 T cells are first activated, then infected with a GFP-expressing strain of HIV (HIV-GFP). Actively infected cells are then flow sorted to obtain a pure infected population (GFP +). These cells are then cultured for three weeks, during which time viral gene expression is progressively downregulated and a latently infected (GFP-) population emerges, although residual GFP expression remains for a subset of the infected cells. When we examined the level of baseline viral gene expression across the different T cell subsets, we observed that Tn and Tcm exhibited a lower level of baseline HIV expression than Tem and Teff cells (21.6% and 37% v 65.8% and 48.9%). Notably, expression of HIV was not affected by the presence of THC at any concentration in any of the CD4 T cell subsets (Fig. 1D). We also examined the impact of THC on the subset markers, CD38 expression and viral GFP expression at an earlier timepoint (6 h) and found no impact of THC on any of these parameters at any THC concentration (Figure S2). Overall, these data indicate that, in this ex vivo latency model system, THC does not have a major impact on the maturation status, activation status or baseline HIV expression in infected CD4 T cells.

### Reactivation of HIV from latency by latency reversing agents is not impacted by THC

**Fig. 2:**
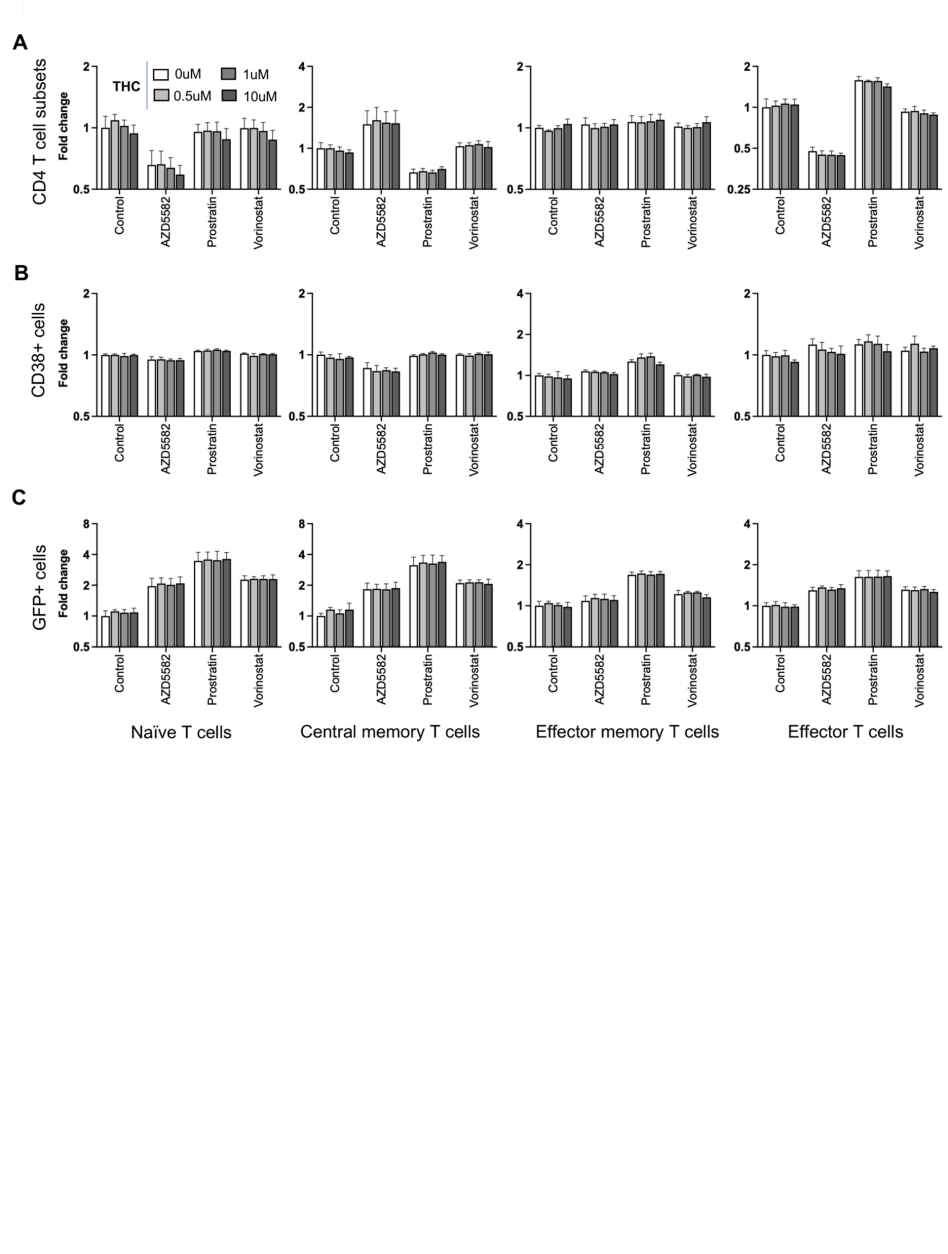
Impact of THC on viral reactivation by latency reversing agents. To assess the impact of THC exposure on reactivation of latently infected CD4 T cells by latency reversing agents (LRAs), we stimulated latently infected CD4 T cells with THC (0, 0.5, 1 and 10 μM) for 24 h followed by a 24 h exposure to one of the three different LRAs with different mechanisms of action—AZD5582 (non-canonical NF-κB agonist), prostratin (PKC agonist), and vorinostat (HDAC inhibitor) or control vehicle (DMSO). **A** Abundance (fold change) of CD4 T cell subsets (Tn, Tcm, Tem, Teff) exposed to THC followed by LRAs stimulation. **B** Expression level (fold change) of CD38 is shown for latently infected CD4 T cell subsets (Tn, Tcm, Tem, and Teff cells) exposed to THC followed by LRAs stimulation. **C** Expression level (fold change) of GFP is shown for latently infected CD4 T cell subsets (Tn, Tcm, Tem, and Teff cells) exposed to THC followed by LRAs stimulation. None of the bars/sample reached statistical significance compared to control vehicle

A major approach in eliminating latently infected cells from PWH on ART involves the pharmacological reactivation of HIV expression with small molecules referred to as latency reversing agents (LRAs). To assess the impact of THC exposure on reactivation of latently infected CD4 T cells by LRAs, we stimulated latently infected CD4 T cells with THC (0, 0.5, 1 and 10 μM) for 24 h followed by a 24 h exposure to one of three different LRAs with different mechanisms of action—AZD5582 (non-canonical NF-κB agonist), prostratin (PKC agonist), and vorinostat (HDAC inhibitor) or control vehicle (DMSO). The cells were then analyzed by flow cytometry to measure expression of CD4 T cell subset surface markers (CCR7, CD45RA, CD38), and viral gene expression (GFP). To facilitate analysis and comparison across cell types, datasets were converted into fold change values normalized to the control vehicle condition. First, we compared the relative frequencies of the CD4 T cell subsets (Tn, Tcm, Tem and Teff) following LRA stimulation with THC exposure. Interestingly, we noticed that some of the LRAs affected the expression of the CD4 T cell subset markers (Fig. 2A). Specifically, we observed that AZD5582 increased the frequency of cells with a Tcm phenotype and decreased the proportion of cells with a Tn or Teff phenotype, while prostratin exposure decreased Tcm cells and increased Teff cells. By contrast, vorinostat had no impact on the expression of subset surface markers. As before, we found that THC exposure had no effect on the frequencies of the different T cell subsets in any of the LRA stimulated conditions. Next, we sought to examine the impact of combined LRA and THC exposure on expression of CD38 in latently infected CD4 T cell subsets (Fig. 2B). LRAs exposure did not strongly affect CD38 expression for most CD4 T cell subsets, although we observed a modest decrease for CD38 expression in Tcm cells exposed to AZD5582, and a modest increase in CD38 for Tem cells exposed to prostratin. When we compared expression of CD38 across the different THC concentrations and LRA conditions, we observed no impact of THC at any concentration in any cell subset. Thus, THC does not affect CD4 T cell subset marker or CD38 expression in the presence or absence of LRAs.

Next, we examined HIV reactivation by LRAs in different CD4 T cell subsets in the presence of THC. After stimulation with the LRAs, we observed that Tn and Tcm exhibited the highest fold change increase in GFP + cells over baseline, while LRAs responses in Tem and Teff were lower, likely due to their lower level of restriction to HIV expression (Fig. 2C). Overall, prostratin was consistently the most potent LRA in all of the CD4 T cell subsets. We observed that THC exposure had no impact on latency reversal for any of the LRAs in any of the CD4 T cell subsets, suggesting that HIV expression and reactivation is robust to THC exposure.

### Integrated single cell RNA- and ATAC-seq profiling of THC stimulated HIV infected cells

Although our data indicated that THC exposure has little impact on latently infected cells in terms of surface marker expression or HIV expression at the protein level, we speculated that THC could have an impact on aspects of the host cells not detectable by these assays. Since most immune cells, including CD4 T cells, express cannabinoid receptors, we hypothesized that THC exposure triggers definable changes to the transcriptome of infected cells that could impact their biological properties.

**Fig. 3:**
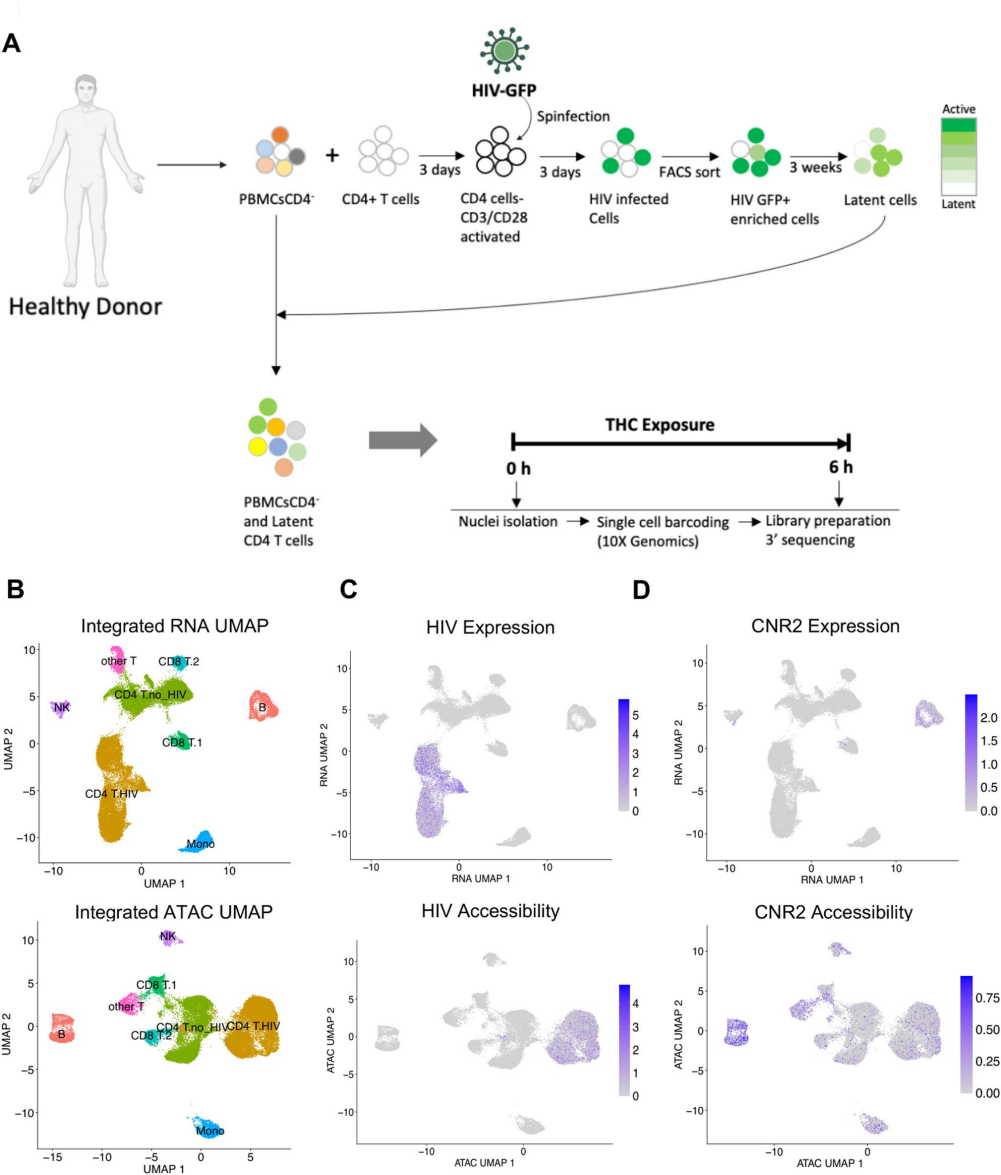
Single cell multiomic analysis of latently infected CD4 T cells exposed to THC. **A** Schematic of experimental design. **B** Uniform Manifold Approximation and Projection (UMAP) dimension reduction of scRNAseq (top) and scATACseq (bottom) with cells labeled by immune cell type. **C** UMAP plot of scRNAseq (top) and scATACseq (bottom) data with cells containing HIV mapping RNA or ATAC reads labeled purple. **D** Same as C but with cells containing RNA or ATAC reads from the CNR2 gene labeled purple. Data show represents aggregated data from three independent donors

To investigate this hypothesis, we first modified our experimental design to analyze a mixture of HIV infected CD4 T cells and autologous PBMCs in order to allow cell-to-cell interactions and indirect effects from bystander cells (Fig. 3A). We stimulated a mixture of latently infected CD4 T cells and autologous PBMCs (at a ratio of 1:3) from a single donor with 500 nM THC for 3, 6, and 12 h, and two additional donors with 500 nM THC for 6 h only. To capture transcriptional and chromatin based responses to stimulation, we then profiled these cells using a combined single cell RNAseq/ATACseq approach (Fig. 3A). After quality control and batch effect removal, integrated scRNA-seq/scATAC-seq yielded a total of 18,407 cells for pre- and 20,956 cells for post-THC exposure with detectable expression of ~ 15,000 genes and ~ 110,700 accessible chromatin peaks detected across the cell population.

Dimension reduction using Uniform Manifold Approximation and Projection (UMAP) of both the scRNA-seq and scATAC-seq data showed several distinct clusters of immune cells (Fig. 3B). To annotate the cell types of the clusters, we used a generalized linear model (GLM)-based cell mapping approach with cell-type “marker” genes curated from the available literature. Briefly, we selected a reference gene panel based on known cell-type-specific gene profiles, then used GLM to test the association of gene expression in each cell with the known marker genes (Fig. 3B). Through the expression of marker genes, each cluster was assigned to a cell type based on the highest percentage of significant cells. This approach deconvoluted the 39,363 cells into specific cell subtypes: B cells, NK cells, Monocytes, CD4 and CD8 T cells. CD4 and CD8 T cells were divided into two separate clusters each, with one cluster likely reflecting the cells that were activated, infected and returned to rest (“CD4 T HIV”) and other being the cells that were added back to the culture after three weeks as part of the PBMC population (“CD4 T no HIV”). CD8 T cells were also present as two major clusters (“CD8T1” and “CD8 T2”), possibly due to CD8 T cells that contaminated the CD4 T cell population that was activated and infected. Another subpopulation of T cells remained unclassified (“other T cells”).

Next, we examined the distribution of viral RNA and ATAC reads across the clusters. As expected, HIV-mapping reads were found largely within only one of the two CD4 T cell cluster sets (CD4 T HIV) for both viral RNA and ATAC reads, allowing us to identify the CD4 T cells derived from the HIV infected culture (Fig. 3C). These data demonstrate that we can identify cells infected with HIV from the presence of reads that map to the viral genome. We also examined the expression and accessibility pattern for the CNR2 gene which encodes the CB2 THC receptor. Interestingly, at the RNA levels CNR2 expression was highest in B cells, while the CNR2 gene was accessible across most immune cell types (Fig. 3D).

### Impact of THC on viral transcription and proviral accessibility

**Fig. 4:**
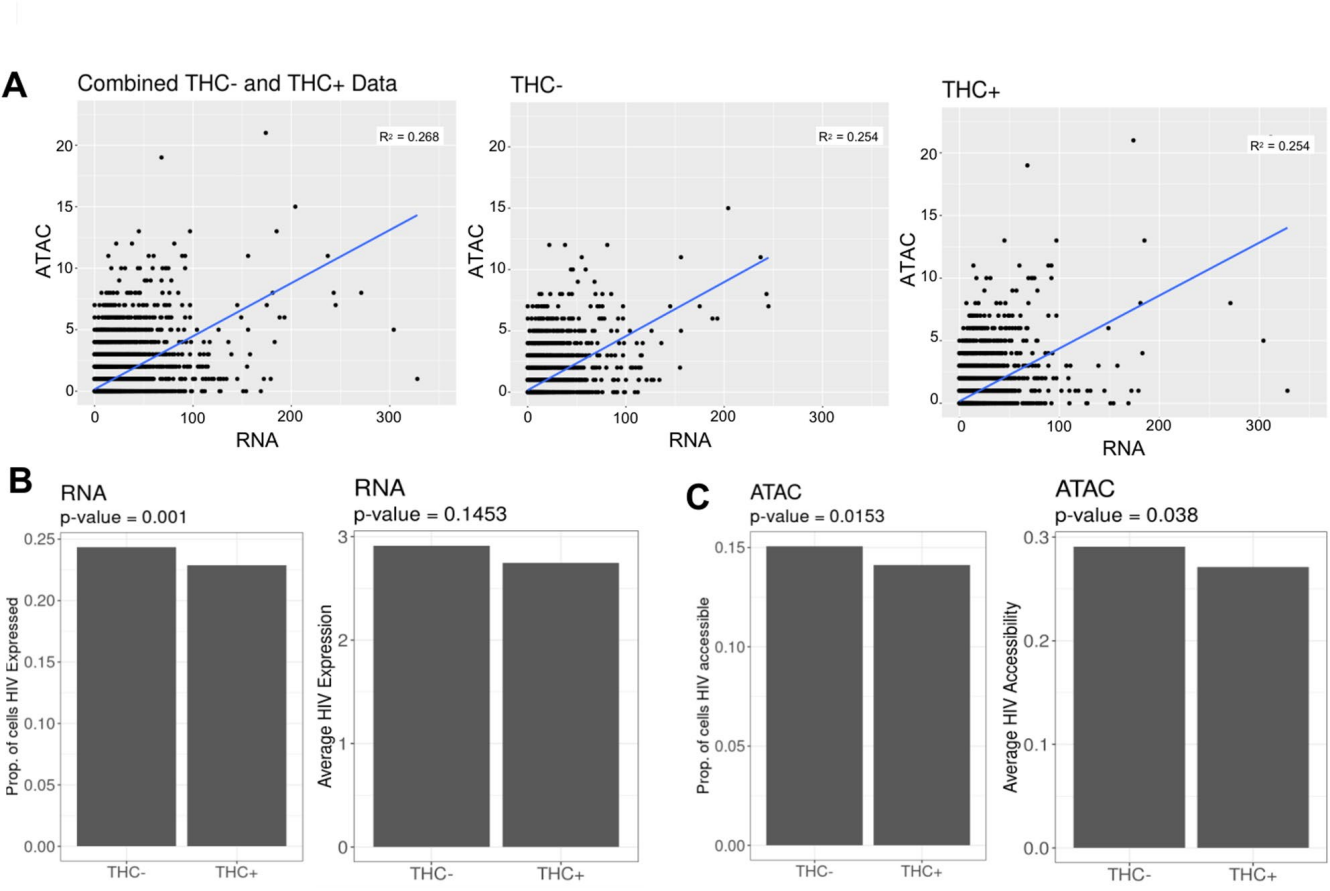
HIV vRNA expression and chromatin accessibility is mildly affected by THC exposure. **A** Correlation scatter plot showing the square root (sqrt) proportion of scRNAseq reads mapping to HIV (X-axis) vs. the sqrt proportion of scATACseq reads mapping to HIV (Y-axis) across the cell population for the integrated data (Left panel), without THC exposure (Middle panel), and with THC exposure (Right panel) for 6 h. **B** Transformed HIV expression data (proportion of cells with HIV expression, Left panel; and Average HIV, Right panel) for cells without and with exposure to THC. **C** Transformed HIV ATAC accessibility data (proportion of cells accessible to HIV, Left panel; and Average HIV accessibility, Right panel) for cells without and with exposure to THC. A permutation non-parametric statistical method were used to assess the significance

We next examined the impact of THC exposure on the HIV proviruses in infected cells at the level of viral transcription and proviral accessibility. During transcriptional activation, pioneer transcription factors recruit chromatin remodeling complexes to the regulatory regions, resulting in increased accessibility at these regions, and these changes in chromatin accessibility are known to play a crucial role in transcription (Tsompana and Buck 2014). The combined scRNAseq/scATACseq approach thus allows us to examine the relationship between changes in accessibility and expression for individual genes. For each cell, we quantified the number of viral RNA (vRNA) reads and viral ATAC (vATAC) reads and we examined the correlation between vRNA and vATAC reads across the cell population (Fig. 4A). Consistent with our previous findings (Ashokkumar et al. 2024), we observed a significant positive correlation between the frequency of vRNA and vATAC reads within the cells, and this relationship was also true for both THC-stimulated and unstimulated cells alone (Fig. 4A). We examined the pattern of vRNA and vATAC reads across the CD4 T HIV UMAP plot, we observed that THC exposure did not noticeably change the pattern of reads distribution for either vRNA or vATAC reads (Figure S3). We then examined the proportion of CD4 T cells with vRNA and vATAC reads as well as the average abundance of vRNA (Fig. 4B) and vATAC (Fig. 4C) reads across the population. Interestingly, we observed that the scRNA-seq and scATAC-seq reads mapping to the HIV genome showed a small but statistically significant reduction in vRNA expression and chromatin accessibility for the HIV infected CD4 T cell population exposed to THC. When we examined the timecourse data from the single donor with multiple timepoints, this effect was visible at all timepoints but was most pronounced at 6 h (Figure S3C). Thus, THC may have a small inhibitory effect on HIV transcription in latently infected cells. Nevertheless, we conclude that, overall, THC has only a minor impact on HIV expression and accessibility in latently infected cells, consistent with our observations by flow cytometry.

### THC exposure modulates infected host cell transcription

Although we observed only a minor impact of THC exposure on HIV expression and accessibility, we hypothesized that THC could impact the transcriptomic or epigenomic phenotype of infected cells as well as uninfected cells within the culture. In addition to allowing us to identify genes whose expression is affected by a given stimulus, the combined scRNA-seq/scATAC-seq approach allows us to quantify the activity of cellular transcription factors (TFs) within each cell based on the enrichment of TF binding sites within accessible chromatin regions.

**Fig. 5:**
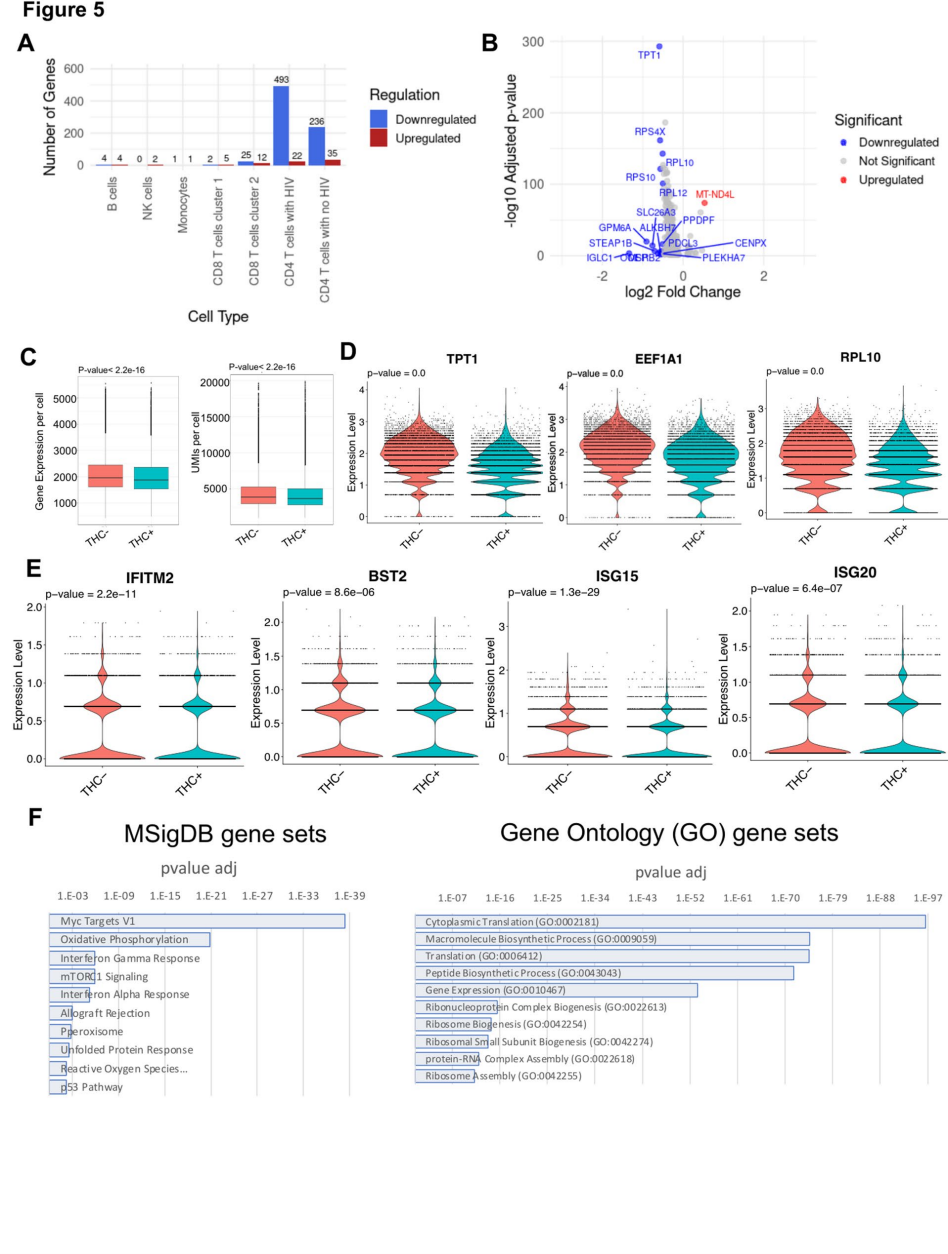
THC affects gene expression in HIV infected CD4 T cells. **A** Bar plot showing the number of differentially expressed genes (DEGs) across different immune cell types. **B** Volcano plot of overall differentially expressed genes (DEGs) between HIV-1 infected CD4 T cells exposed to THC vs non-exposure. The significance in DEGs were set with the log2 fold change threshold <—0.5 and the adjusted *p*-value threshold <—0.05. **C** Box plot showing the average expressed genes (Left panel) and UMIs (Right panel) per cell by condition. **D** Violin plot showing downregulation of selected genes that contribute to protein translation, including genes encoding ribosomal subunits (TPT1, EEF1A1, and RPL10) in response to THC exposure. **E** Violin plot showing expression of selected interferon sensitive genes (IFITM2, ISG15, ISG20 and BST2) in THC-stimulated HIV infected cells. *P* values from Wilcoxon rank sum tests comparing control and THC exposure. **F** MSigDB gene set enrichment (Left panel) and Gene ontology analysis of downregulated DEGs for HIV infected cells after THC exposure

To maximize our ability to identify THC-induced changes in gene expression and TF activity, we considered DEGs within specific subsets of cells (B cells, NK cells, monocytes, CD4 T, and CD8 T cells). For CD4 T cells we also separately considered cells from the HIV infected cluster and the uninfected (PBMC derived) cluster. Across the different immune cell types, we observed considerable variation in the magnitude of the transcriptomic response to THC (Fig. 5A, Table S1). In most cell subsets, we observed a small number of DEGs, with 15 in B cells (11 up, four down), two in NK cells, two in monocytes, seven in CD8T1 cells and 37 in CD8T2 cells. By contrast, CD4 T cells exhibited a more pronounced THC response, with 271 DEGs in the uninfected CD4 T cell cluster and 515 DEGs in the HIV infected CD4 T cell cluster. A previous study also identified DEGs in PBMCs from healthy donors administered with THC for 70 min (Hu et al. 2020). Notably, the DEGs detected in CD4 T cells in our study were largely distinct from those reported in this prior study (Hu et al. 2020) likely reflecting the different cellular environment in an in vitro model system. Notably, the changes in gene expression for both the CD4 T cell clusters were highly polarized, with 35 upregulated and 236 downregulated genes in uninfected CD4 T cells, and 22 upregulated and 493 downregulated genes in the infected cells (Fig. 5A, B). Overall, we identified an average of 2,017 expressed genes in THC-exposed cells compared to 2,097 genes in the non-exposed control group (Fig. 5C, Left panel). This was accompanied by a slight reduction in total transcript counts, with an average of 4233 UMIs in the THC + group compared to 4414 UMIs in the control (Fig. 5C, Right panel). These metrics indicate a modest downward shift in total transcriptional output in THC-exposed HIV infected CD4 T cells, and suggest that THC exposure has an overall suppressive effect on gene expression in CD4 T cells.

Several noteworthy genes were observed in the set of differentially expressed genes (Table S1). In B cells and in uninfected CD4 T cells, we observed downregulation of the pro-inflammatory cytokine IL-1β, consistent with previous reports that CB signaling inhibits inflammasome activation (Suryavanshi et al. 2020). In THC stimulated B cells, CD8 T cells and uninfected CD4 T cells, we observed strong downregulation of the ion transporter SLC26A3 that regulated intestinal permeability. Within both infected and uninfected CD4 T cells, the set of downregulated DEGs contained numerous genes that contribute to protein translation, including genes encoding ribosomal subunits, suggesting a negative impact of THC on overall cell metabolism and protein synthesis in infected cells (Fig. 5D). Interestingly, we also observed that several known interferon stimulating genes (ISGs) were downregulated in THC-stimulated HIV infected cells including IFITM2, ISG15, ISG20 and BST2 (Fig. 5E). Notably, BST2 has been identified as a key HIV restriction factor that inhibits HIV particle release and is counteracted by the viral protein, Vpu (Jia et al. 2014; Mi et al. 2015). These data suggest that THC exposure could contribute to suppressing the expression of innate antiviral pathways in infected cells.

We then examined enrichment of specific biological pathways in the sets of DEGs for uninfected and infected CD4 T cells. Specifically, we examined the DEGs for enrichment with curated sets of pathway-associated genes using ENRICHR (Kuleshov et al. 2016). For both infected and uninfected CD4 T cells, no specific pathways were found to be enriched in the set of THC-upregulated genes, likely due to the small numbers of upregulated DEGs. By contrast, in the set of genes that were downregulated in uninfected CD4 T cells, we observed significant enrichment for four gene sets within MSigDB—Myc target genes, TNF-alpha Signaling via NF-κB, p53 Pathway, and Allograft Rejection (Table S2). In infected cells, 19 pathways were enriched, including Myc Targets, mTORC1 signaling and oxidative phosphorylation (Fig. 5F left panel, Table S3). For the GO Biological Process sets, Cytoplasmic translation (GO:0002181) and Macromolecule Biosynthetic Process (GO:0009059) were the top two enriched gene sets in the downregulated DEGs for both infected and uninfected cells, consistent with the hypothesis that THC has a negative effect on protein synthesis and metabolism for both infected and uninfected cells (Fig. 5F right panel, Table S4, Table S5).

### THC exposure affects the activity of specific TFs in HIV infected CD4 T cells

We next examined the impact of THC exposure on the activity of transcription factors within the different immune cell populations. During transcriptional activation, pioneer transcription factors bind to DNA in a sequence-specific manner and recruit chromatin remodeling complexes that promote increased accessibility within key gene controlling regions (Tsompana and Buck 2014). Since scATAC-seq data provides genome-wide accessibility information from each cell, we can thus estimate the activity of cellular transcription factors within each cell from these data. For each cell in the population, a TF activity score was calculated for a set of 600 cellular TFs with known binding site sequences, based on the enrichment of these sequences within the open chromatin in that cell. Using this TF activity matrix we then identified differentially active transcription factors (DATFs) that exhibited altered activity after THC stimulation (Table S6). To further characterize the epigenetic impact of THC, we performed a differential accessibility analysis to identify significant differentially accessible regions (DARs) across all immune cell clusters. Our analysis revealed that THC stimulation significantly increased chromatin accessibility within infected CD4 T cells (Table S7, Figure S4), demonstrating that THC exposure elicits a remodeling of the chromatin landscape.

**Fig. 6:**
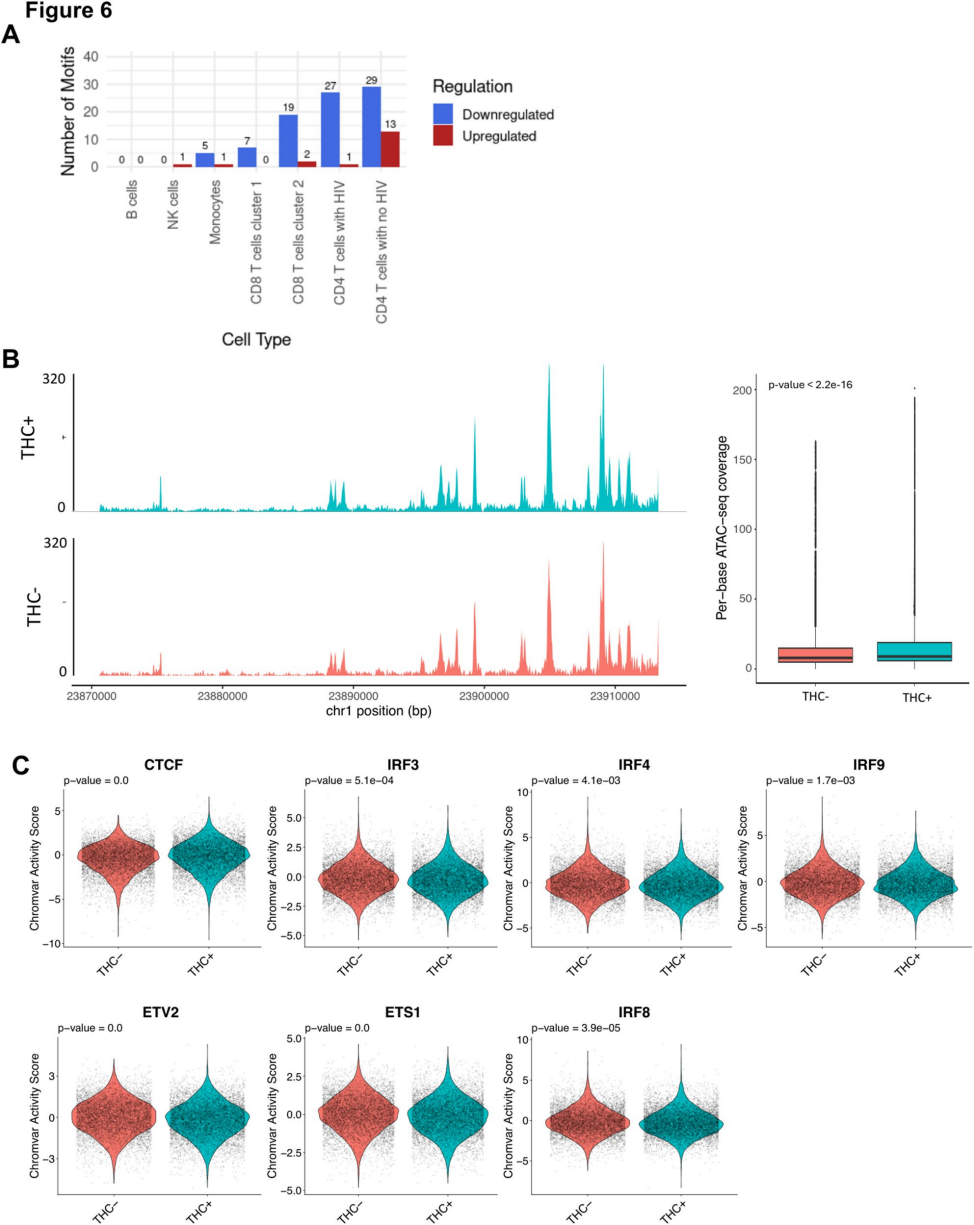
THC impacts transcription factor activity in HIV infected CD4 T cells. **A** Bar plot showing the number of differentially active transcription factors (DATFs) across the different immune cell types after 6 h THC exposure. **B** Representative scATAC-seq peaks at the CNR2 region on genome visualization tracks (Left panel, and the bar graph showing the average read counts of CNR2 accessibility (Right panel). **C** Violin plots showing activity scores for selected TFs (CTCF, IRF3, IRF4, IRF9, ETV2, ETS1, IRF8) in response to THC exposure. *P* values from Wilcoxon rank sum tests comparing THC treated cells to control treated cells

Similar to our observations with differentially expressed genes (DEGs), we observed that the impact of THC was highly variable depending on the immune cell type (Fig. 6A, Table S6). To validate the direct impact of THC on its primary receptor, we first examined the Tn5 transposase accessibility at the CNR2 gene (Fig. 6B). Interestingly, our analysis revealed that THC exposure significantly enhanced chromatin accessibility within the CNR2 gene region in latently infected cells. Specifically, we observed a measurable increase in signal intensity, with cells exposed to THC exhibiting an average of 17.4 reads per base, compared to 14.3 reads per base in non-exposed control cells. This enrichment confirms that THC treatment promotes a more open and accessible chromatin state at the CNR2 gene.

Next, we investigated THC regulated DATFs in different immune cell types. Surprisingly, no DATFs were identified in B cells, despite high expression of CNR2 in these cells. Six THC-responsive DATFs were identified in monocytes – one was upregulated (FIGLA) and five were downregulated (GABPA, ELF1, ELF3, EHF, and IKZF1). Notably, all of the downregulated DATFs except IKZF1 are members of the ETS TF family. Within NK cells, a single DATF was identified, the transcriptional regulator CTCF, whose activity was increased in NK cells in response to THC exposure. In CD8 T cells, we observed 7 DATFs, all which were downregulated and, notably, all of these DATFs were members of ETS family.

Consistent with our DEG analysis, we observed that the strongest impact of THC was within CD4 T cells. Within uninfected CD4 T cells, we observed 42 DATFs—13 upregulated and 29 downregulated. Similar to our observations with monocytes, the downregulated DATFs were largely composed of ETS TFs, but also included IKZF1. Within the set of upregulated DATFs, CTCF was the most highly upregulated, although two TFs that have been shown to regulate HIV expression, ATF4 and YY1, were also upregulated. Within HIV infected CD4 T cells, we observed 28 DATFs, only one of which (CTCF) was upregulated. Similar to what we had observed for uninfected CD4 T cells, the majority of the 27 downregulated TFs were ETS family members (ETV2, ETS1, ERG, FLI1, ELF4, ELK3, ERF, FEV, ETV3, ETS2, ELF5, EHF, ETV5, ELF2, ETV6, ELK4, ELF3, GABPA, ELF1, ELK1, ETV1), while IKZF1 was also downregulated. Interestingly, however, we also observed that there were three interferon regulated TFs (IRF3, IRF8 and IRF9) that were downregulated in infected CD4 T cells (Fig. 6C, Table S6), but not in uninfected CD4 T cells. This finding is consistent with our observation that ISGs were transcriptionally downregulated in HIV infected CD4 T cells. In addition, we performed footprinting analysis for the significant DATFs we identified in the CD4 T cell cluster with HIV, but did not observe a clear difference in binding site occupancy (Figure S5). Overall, these data indicate that THC exposure may affect the activity of several TFs in HIV infected CD4 T cells. In particular, we observed that CTCF activity was upregulated while ETS TFs and IRFs were downregulated in THC exposed HIV infected CD4 T cells. These altered patterns of TF activity likely explain the altered gene expression in these cells after THC exposure.

## Discussion

Cannabis (CB) use is common in people with HIV (PWH) and CB exposure may exert potential therapeutic benefits to PWH due to its anti-inflammatory properties (Maggirwar and Khalsa 2021; Yin et al. 2022). It was previously reported that cannabis use is associated with a lower rate of neurocognitive impairment (Watson et al. 2020) and lower risk of progression to AIDS (Coates et al. 1990) in PWH. CB consists of a heterogeneous mixture of compounds, but the primary cannabinoid constituent is Δ−9-tetrahydrocannabinol (THC). THC has been shown to suppresses expression of pro-inflammatory cytokines and thereby reduces systemic immune activation in HIV infection (Mboumba Bouassa et al. 2023), but THC's impact on the latent HIV reservoir is unclear. However, recently published reports have indicated that reservoir size could be affected by CB use. Our lab recently showed, using a cohort of CB using PWH on ART, the CB use is associated with altered abundances of T cell subsets as well as a trend towards a smaller intact HIV reservoir (Falcinelli et al. 2023). Another study also recently reported that CB use was associated with lower tissue reservoir burden in PWH with clade C infections (Liu et al. 2024).

In this present study, we have analyzed the impact of THC on HIV infection using a primary CD4 T cell model of HIV latency. Overall, we find that HIV expression and reactivation by LRAs are largely unaffected by THC exposure over a time period of 24 h. Similarly, we saw no significant impact of THC on expression of the activation marker CD38 or on the expression of surface markers use to describe CD4 T cell memory subsets. This observation was robust across a wide range of THC concentrations. Nevertheless, it remains possible that a longer term or repeated exposure could influence HIV expression or LRA sensitivity. THC sensitivity of infected cells could also be modulated by the more complex environment in vivo, either in peripheral blood or in solid tissues such as the gut or brain. Animal models of HIV infection and CB exposure may thus be necessary to fully evaluate the impact of THC on HIV expression and reactivation.

In addition to measuring the impact of THC on HIV expression, we carried out an integrated single cell multiomic analysis of genome-wide chromatin accessibility and gene expression to characterize latently infected CD4 T cells exposed to THC. While these data showed a small reduction in viral RNA and proviral accessibility after 6 h of THC exposure, we observed a number of changes to gene expression and TF activity in HIV infected CD4 T cells. In particular, we observed a strong transcriptional downregulation of genes that contribute to protein synthesis, as well as interferon-stimulated genes. This observation suggests that THC represses macromolecular metabolism and protein expression in infected cells, which could affect the ability of infected cells to respond to antigen or cytokine cues. This finding thus could help to explain why CB users may have a smaller HIV reservoir size than non-users – if periodic T cell activation is necessary for intermittent clonal expansion that sustains the reservoir, THC suppression of T cell activation and metabolism could lead to a lower rate of reservoir clonal expansion over time, leading to more rapid reservoir depletion. The reduced expression of ISGs in infected cells also merits further investigation. The reduced activity of an innate sensing pathway in infected cells could contribute to the overall lower level of immune activation and exhaustion in PWH who use CB. The precise innate pathway that is active in infected cells and the molecular mechanism by which THC blocks this pathway will need to be elucidated.

Our analysis of differentially active transcription factors in HIV infected CD4 T cells after THC stimulation also produced some intriguing observations. Specifically, we observed that THC-induced increased binding activity of the transcriptional regulator CTCF. CTCF is a chromatin-binding protein that mediates long-range chromatin looping as part of the cohesin complex (Zhou et al. 2022). CTCF has also been shown to silence the HIV promoter by inducing repressive chromatin structures (Jefferys et al. 2021). We also observed a strong enrichment of ETS TFs within the set of TFs with downregulated activity after THC exposure. Notably, ETS1 has been identified as a master regulator of ribosomal gene expression (Xiao et al. 2022), suggesting that reduced activity of ETS transcription factors likely contribute to the reduced expression of ribosomal genes after THC exposure. We have also recently identified ETS1 as a key mediator of HIV repression in resting CD4 T cells (Ashokkumar et al. 2025). ETS family of transcription factors can act as activators or repressors of gene expression (Ashokkumar et al. 2025). THC exposure affects MAPK signaling pathways (Simon et al. 2016), which can regulate ETS family activity. For instance, the ERK pathway influenced by THC can modulate ETS1 or ELK3 activity, potentially affecting HIV latency dynamics. In addition to CTCF and ETS family of TF motifs, we also identified downregulated activity of Interferon regulator factor (IRF) proteins (IRF3, IRF4, IRF8, IRF9) in THC exposed HIV infected cells. Notably, this downregulation was not observed in uninfected CD4 T cells, suggesting that this phenomenon is specific to HIV infected cells. IRFs play key roles in the activation of ISGs in response to viral infection, consistent with our observation of reduced ISG expression in THC exposed cells. Further work to clarify the role of specific ETS transcription factors and IRFs in the response to THC in infected cells will be required.

Our analysis revealed a significant number of DEGs and DATFs in THC-treated CD4 T cells despite the apparently low expression of CNR2 RNA in this cell type. While the basis for this finding is not fully understood, we can potentially reconcile it by considering the nature of the cannabinoid receptor type 2 (CB2), encoded by the CNR2 gene. As a G protein-coupled receptor (GPCR), CB2 is well-known for its potent signal amplification properties, which may vary between cell types. This process allows a small number of activated receptors to trigger a large intracellular cascade, including the production of second messengers and the activation of kinases. This cascade exponentially amplifies the initial signal, enabling a limited number of receptors to elicit a widespread biological response (Arshavsky and Burns 2014; Zhang et al. 2024). Therefore, we hypothesize that the high number of DEGs and DATFs observed is a direct result of a robust, cell-type-specific signal amplification mechanism.

Our findings should be considered in the light of several caveats and limitations to our study. Our approach uses an in vitro model of HIV latency that may not fully reflect the regulation of the clinical reservoir. The activation of CD4 T cells intrinsically skews the distribution of memory phenotypes compared to resting T cells observed in vivo. While our comparison of THC exposed to non-exposed cells robustly identifies the THC-specific effects within this activated population, this model could differ from the natural subset distribution of CD4 T cells *in vivo*. Furthermore, CB is comprised of several cannabinoids other than THC that may play important roles in affecting the latent HIV reservoir and in immune exhaustion in PWH on ART. Confirmation of expression for CB2/CNR2 in CD4 T cells at the protein level has been challenging due to a lack of reliable antibodies against CB2. A broader investigation of the impact of minor cannabinoids on HIV infection could yield important new insights. The transcriptional and epigenomic response of infected cells to THC exposure could also vary depending on the length of exposure.

Nevertheless, our findings reveal some important new details regarding how CB use could impact the HIV reservoir and immune systems of PWH and highlight the value of multiomic approaches to understanding the responses of immune cells to stimuli. In particular, the downregulation of protein synthesis and antiviral pathways in HIV infected cells following THC exposure could be a significant contributor to the documented impact of CB use on the immune systems of PWH. Further research will help to clarify these connections and potentially help to guide HIV cure approaches that are customized for PWH with substance abuse disorder.

## Supplementary Information

Supplementary Material 1.

Supplementary Material 2.

## Data Availability

The data reported in this study have been deposited in Gene Expression Omnibus (GEO: GSE313059, and are publicly accessible at https://www.ncbi.nlm.nih.gov/geo.
