# Supplementary material for: Single cell multiomic analysis of the impact of Delta-9-tetrahydrocannabinol on HIV infected CD4 T cells
Source: J Cannabis Res. 2026 Mar 5;8:52. doi: 10.1186/s42238-026-00412-0 (PMC13069807; doi:10.1186/s42238-026-00412-0)

Supplementary Figure S2

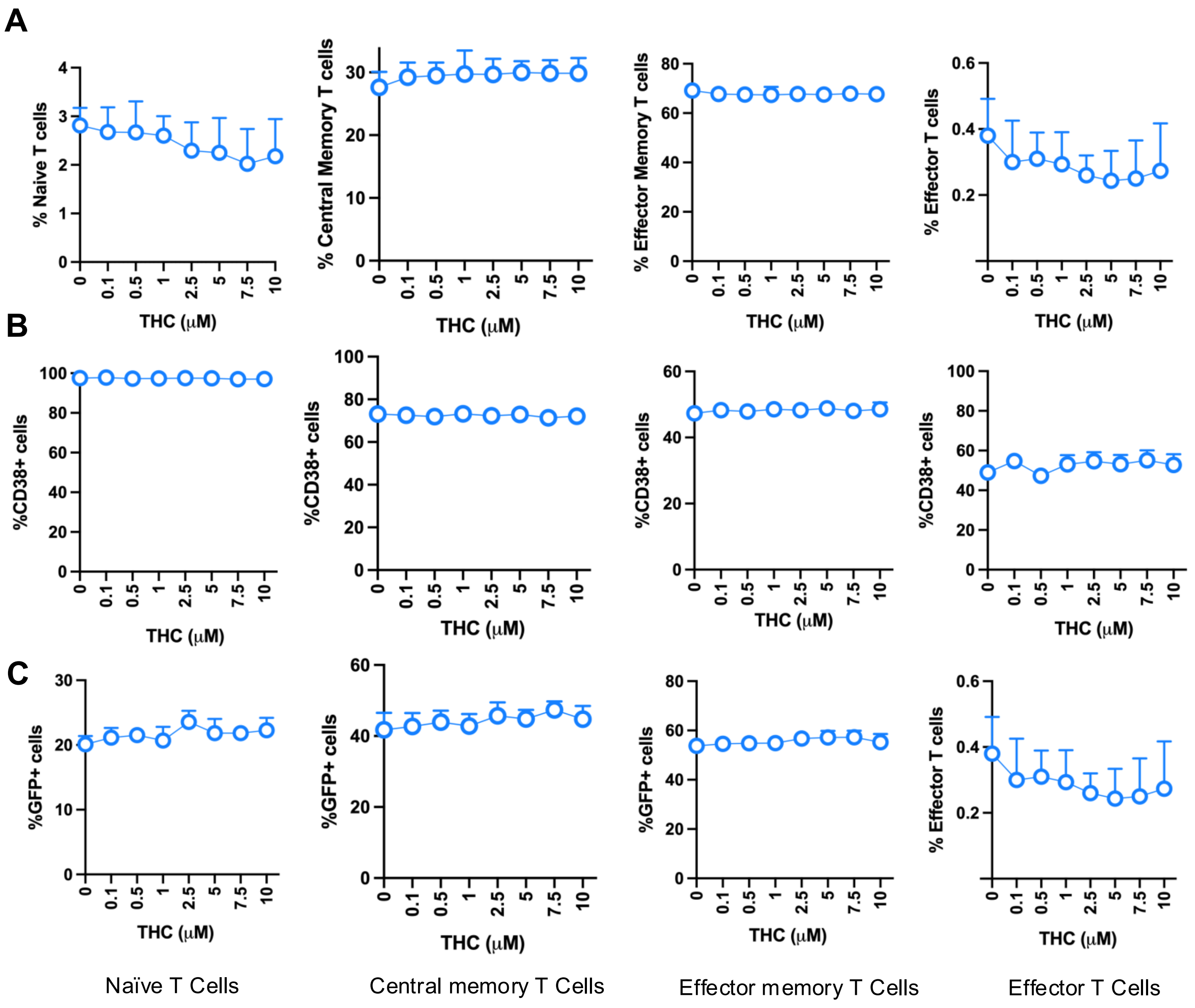

Supplement: Supplementary file 1 — Supplementary Material 1. [file 42238_2026_412_MOESM1_ESM.zip › Figure S2 revised.pdf]
