# Supplementary figures and images for: Single cell multiomic analysis of the impact of Delta-9-tetrahydrocannabinol on HIV infected CD4 T cells
Source: J Cannabis Res. 2026 Mar 5;8:52. doi: 10.1186/s42238-026-00412-0 (PMC13069807; doi:10.1186/s42238-026-00412-0)

# Supplementary Figure S1

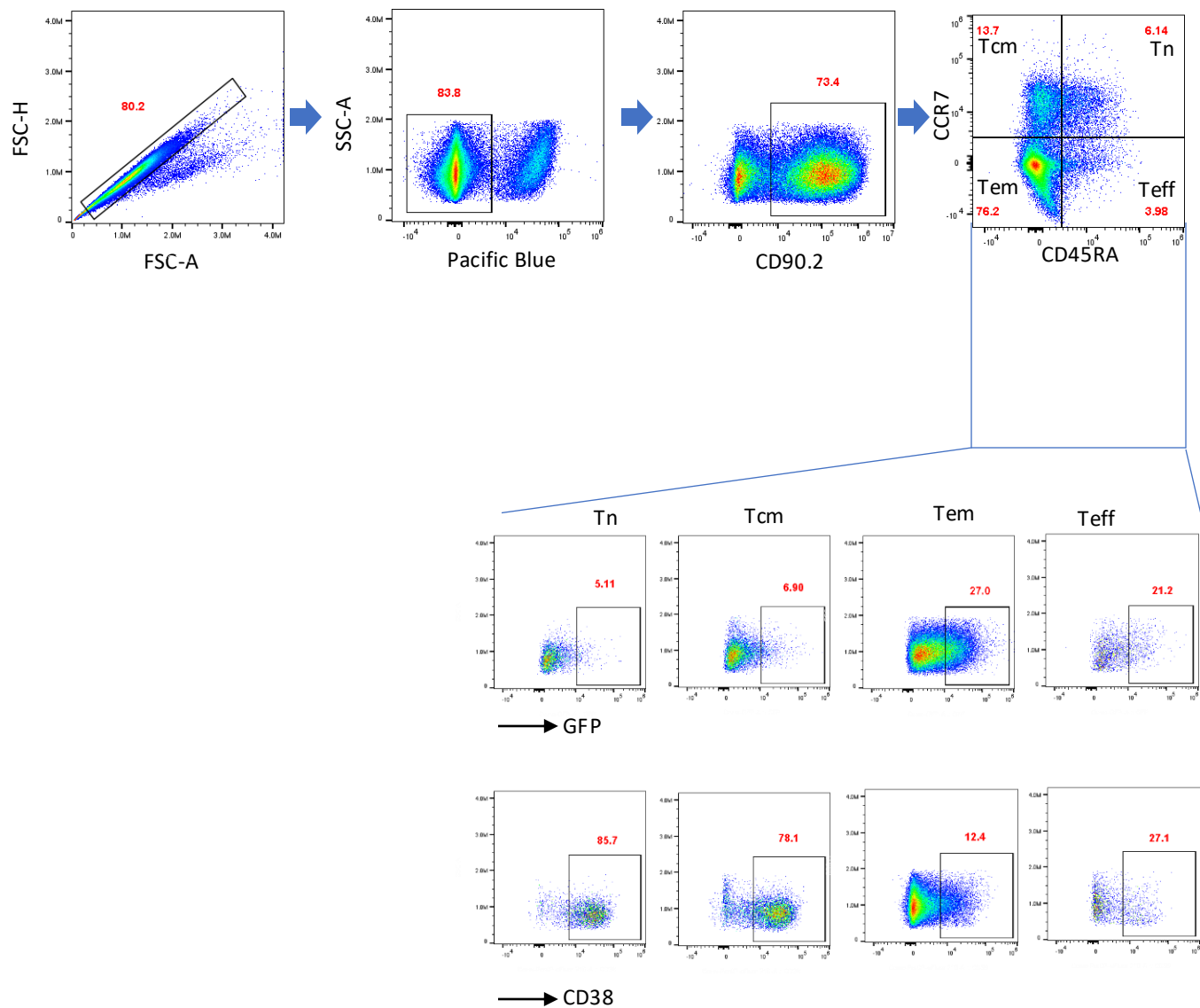

Supplement: Supplementary file 1 — Supplementary Material 1. [file 42238_2026_412_MOESM1_ESM.zip › Figure S1 revised.pdf]

## Slide 1
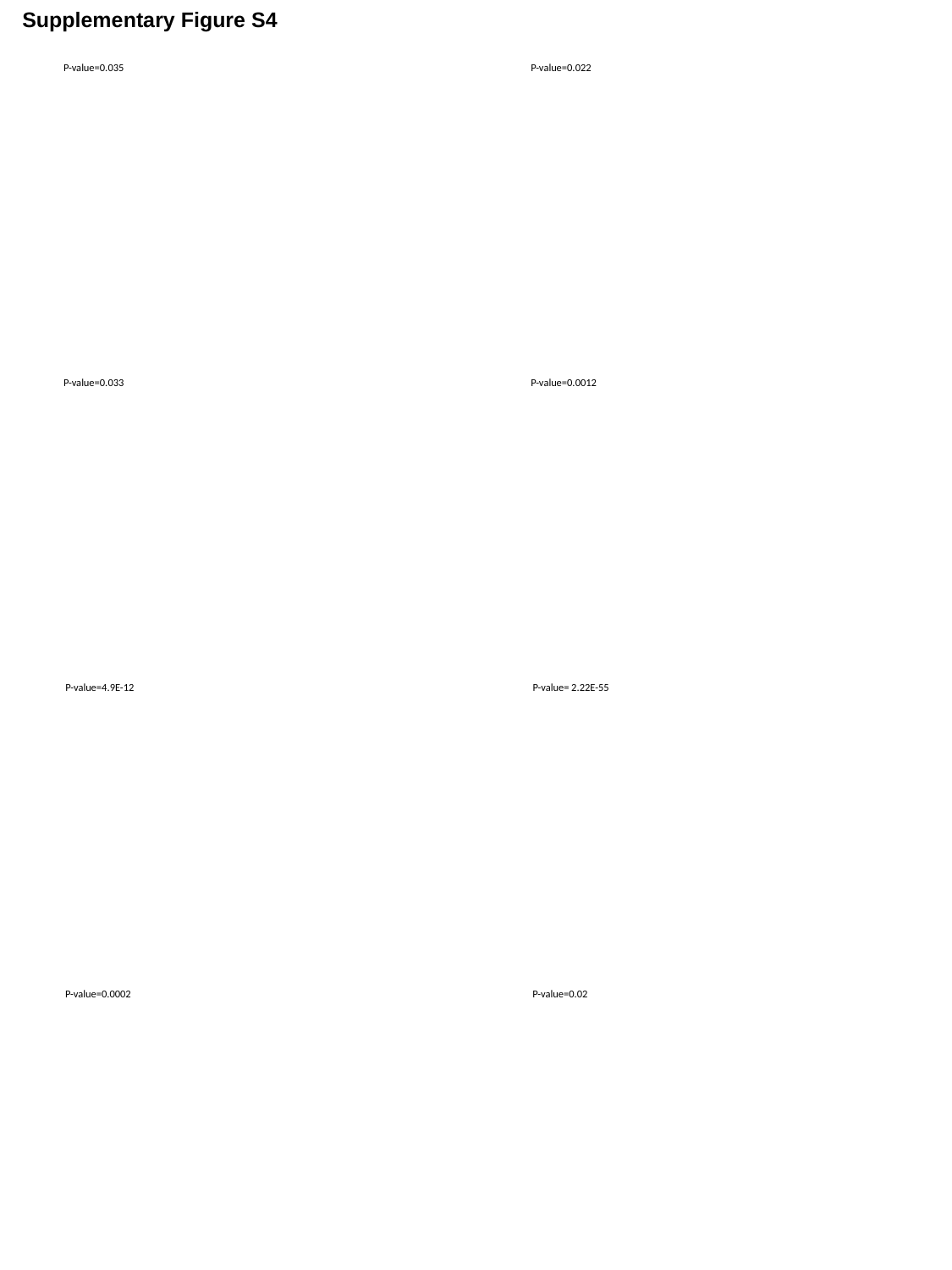

Supplementary Figure S4
P-value=0.035
P-value=0.022
P-value=0.033
P-value=0.0012
P-value=4.9E-12
P-value= 2.22E-55
P-value=0.0002
P-value=0.02

Supplement: Supplementary file 1 — Supplementary Material 1. [file 42238_2026_412_MOESM1_ESM.zip › Figure S4.pptx]

Supplementary Figure S3

**A**

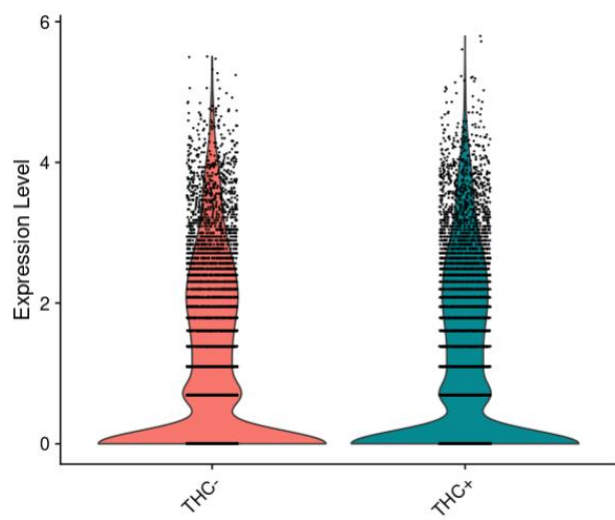

**B**

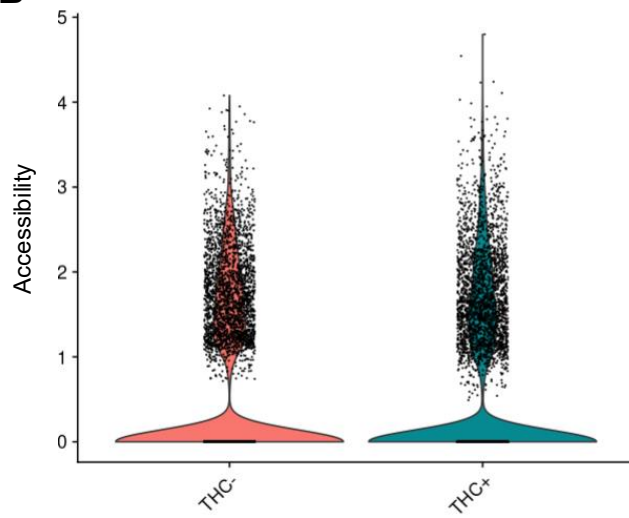

Supplement: Supplementary file 1 — Supplementary Material 1. [file 42238_2026_412_MOESM1_ESM.zip › Figure S3 revised.pdf]
